# Evaluation of the Teaching Recovery Techniques community-based intervention for unaccompanied refugee youth experiencing post-traumatic stress symptoms (Swedish UnaccomPanied yOuth Refugee Trial; SUPpORT): study protocol for a randomised controlled trial

**DOI:** 10.1186/s13063-019-3814-5

**Published:** 2020-01-10

**Authors:** Anna Sarkadi, Georgina Warner, Raziye Salari, Karin Fängström, Natalie Durbeej, Elin Lampa, Zaruhi Baghdasaryan, Fatumo Osman, Sandra Gupta Löfving, Anna Perez Aronsson, Inna Feldman, Filipa Sampaio, Richard Ssegonja, Rachel Calam, Anna Bjärtå, Anna Leiler, Elisabet Rondung, Elisabet Wasteson, Brit Oppedal, Brooks Keeshin

**Affiliations:** 1 Child Health and Parenting (CHAP), Department of Public Health and Caring Sciences, Uppsala University, Box 564, BMC, Husargatan 3, 751 22 Uppsala, Sweden; 2 Division of Clinical Psychology, University of Manchester, Manchester, UK; 3 Department of Psychology, Mid-Sweden University, Östersund, Sweden; 4 Division of Mental Health, Norwegian Institute of Public Health, Oslo, Norway; 5 Department of Pediatrics, University of Utah, Salt Lake City, Utah USA

**Keywords:** Teaching Recovery Techniques, Post-traumatic stress disorder, Unaccompanied refugee minors, Randomised controlled trial

## Abstract

**Background:**

In 2015, 162,877 persons sought asylum in Sweden, 35,369 of whom were unaccompanied refugee minors (URMs). Refugee children, especially URMs, have often experienced traumas and are at significant risk of developing mental health problems, such as symptoms of post-traumatic stress disorder (PTSD), depression and anxiety, which can continue years after resettlement. The Swedish UnaccomPanied yOuth Refugee Trial (SUPpORT) aims to evaluate a community-based intervention, called Teaching Recovery Techniques (TRT), for refugee youth experiencing PTSD symptoms.

**Methods/design:**

A randomised controlled trial will be conducted in which participants will be randomly allocated to one of two possible arms: the intervention arm (*n* = 109) will be offered the TRT programme, and the waitlist-control arm (*n* = 109) will receive services as usual, followed by the TRT programme around 20 weeks later. Outcome data will be collected at three points: pre-intervention (T1), post-intervention (T2; about 8 weeks after randomisation) and follow-up (T3; about 20 weeks after randomisation).

**Discussion:**

This study will provide knowledge about the effect and efficiency of a group intervention for URMs reporting symptoms of PTSD in Sweden.

**Trial registration:**

ISRCTN, ISRCTN47820795. Prospectively registered on 20 December 2018.

## Background

In 2015, 162,877 persons sought asylum in Sweden, 35,369 of whom were unaccompanied refugee minors (URMs) [40]. Most URMs (86%) were boys, mainly from Afghanistan, Syria, Somalia and Eritrea. The number of new applications has dropped sharply since 2015; however, Sweden is still one of the main destinations for URMs, and many of the minors who arrived in 2015 still remain in the country. Apart from adverse events before and during migration, the asylum and resettlement process per se involves stressors, such as lack of control and insecurity while waiting for a decision of the asylum application. Both pre- and post-migration factors severely increase the risk of developing mental health problems [32].

Vulnerability is experienced by many immigrants and refugees, yet URMs appear to be the most vulnerable [4]. A study on 307 URMs in Norway showed that 54% reported high levels of post-traumatic stress disorder (PTSD) symptoms [14]. A study of 206 (mainly male Afghani) URMs in Sweden reported that 76% screened positive for PTSD symptoms [27]. Another Swedish study of 42 children from refugee families indicated that 21% met the full criteria for PTSD, and a further 31% suffered from severe PTSD symptoms [1]. The prevalence of PTSD did not decrease at the 2.5-year follow-up [1]. Longitudinal studies of URMs confirm high levels of mental health problems several years after resettlement [20, 43]. Thus, PTSD symptoms are not only prevalent in asylum-seeking children and youth, they also tend to persist [41]. Considering the associations between PTSD and lower academic achievements [44] and unemployment [45], integration might also be aggravated.

There is general agreement in the literature that trauma-focused cognitive behavioural therapy (TF-CBT) is the method of choice for treating PTSD and other internalising and externalising symptoms in trauma-exposed children and youth [15]. In Sweden, although it is the treatment of choice, TF-CBT is unfortunately not equally accessible or available in a timely manner for refugee children and youth due to resource constraints within specialised services. PTSD is associated with other mental health disorders such as anxiety, depression and substance use and, when untreated, it can lead to functional impairment in school and work, as well as increase the risk of suicide. For such a large group within society to experience mental health problems and associated poor health and integration outcomes without receiving the necessary support is unacceptable. There is a need to develop a stepped-care model with light-touch interventions tied into specialised services.

## Teaching Recovery Techniques

The Children and War Foundation, based in the UK and Norway, utilised TF-CBT techniques to develop Teaching Recovery Techniques (TRT) [34, 46]. The brief manualised intervention aims to increase coping and promote recovery from PTSD in children aged eight and above in conflict/disaster. It was specifically designed to meet the needs of low-resource settings, where a lot of children require intervention. High acceptability and large effect sizes for decrease in symptoms of both PTSD and depression have been reported in studies from Gaza [3, 25] and after the tsunami in Thailand [23].

In Sweden, an exploratory trial of TRT with 46 URM youth (mainly male, aged 13–18 years) yielded promising results, with significant decreases in both PTSD and depression reported [28]. More than a fifth of participants recovered from their PTSD symptoms, while a third recovered from depressive symptoms [28].

The Swedish UnaccomPanied yOuth Refugee Trial (SUPpORT) aims to further strengthen the evidence base of TRT among refugee youth (aged 14–20 years for the present project) residing in Sweden. By applying a randomised controlled approach, outcomes can be attributed to the TRT intervention. The present paper outlines the protocol for SUPpORT.

## Objectives

The objectives of the trial are:

1. To evaluate whether the TRT programme has a positive effect on unaccompanied refugee youth mental health in comparison to similar youth who only receive services as usual
2. To evaluate whether the TRT programme has a positive effect on unaccompanied refugee youth self-efficacy and well-being, which relate to the programme theory of change
3. To identify which subgroups report the most/least benefit of TRT
4. To describe the extent to which TRT is implemented with fidelity to programme design
5. To estimate the cost-utility and cost-effectiveness of the TRT programme.

It is hypothesised that, when compared with youth who have not received the intervention (the waitlist-control arm), youth who have received TRT (the intervention arm) will demonstrate fewer self-reported symptoms of mental ill health, specifically PTSD, depression and anxiety symptoms. It is further hypothesised that, when compared with the waitlist-control arm, the intervention arm will report greater self-efficacy and well-being.

## Methods

### Design

**Fig. 1:**
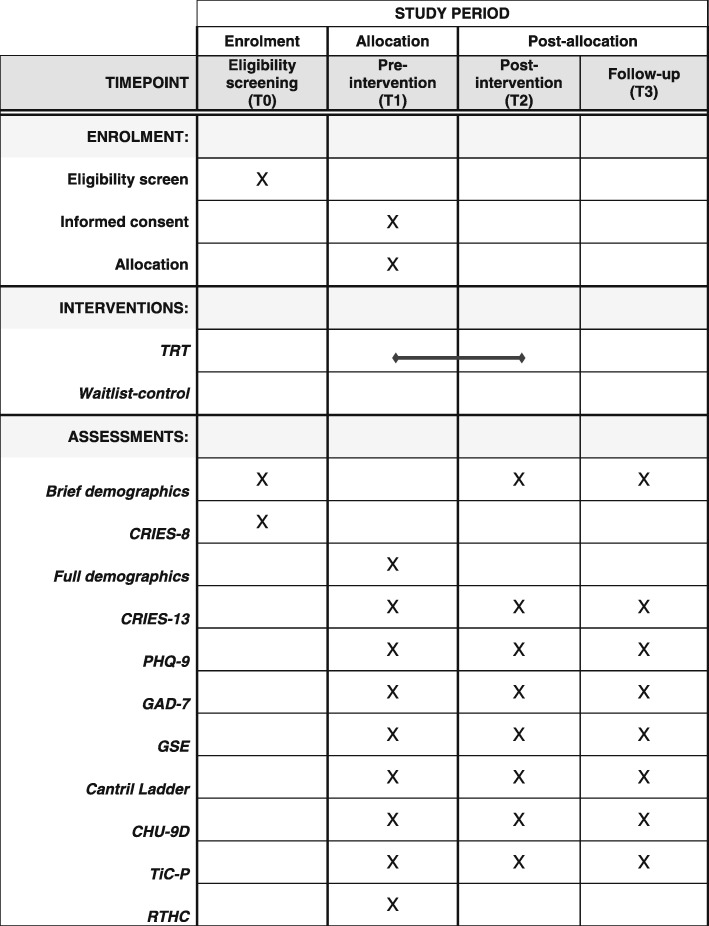
SUPpORT schedule of enrolment, interventions and assessments

A two-arm randomised waitlist-control superiority trial (1:1 allocation ratio) will be conducted to evaluate the effectiveness of the TRT programme in improving mental health outcomes in unaccompanied refugee youth who have self-reported symptoms of PTSD. The intervention arm will be offered the TRT programme immediately after randomisation and the waitlist-control arm around 20 weeks later; both trial arms will have access to services as usual. Assessments will take place at three points: pre-intervention (T1), post-intervention (T2; about 8 weeks after randomisation) and follow-up (T3; about 20 weeks after randomisation). See Fig. 1 for an overview of assessments. The Standard Protocol Items: Recommendations for Interventional Trials (SPIRIT) checklist is presented in Additional file 1.

### Setting

TRT-trained ‘group leaders’ will deliver the intervention, with two group leaders for each group (with the assistance of interpreters, if necessary). The interventions will be delivered in a range of community settings (e.g. health care centres, social service group homes, non-governmental organisations [NGOs]) across Sweden. Sites include both urban and rural municipalities that have accepted to host refugees. To promote intervention stability, it is recommended that each site has an assigned local coordinator and at least one experienced group leader (i.e. one who has conducted at least two previous TRT groups). Supervision will also be offered to TRT group leaders. Assessments for the randomised controlled trial (RCT) will also take place in community settings.

### Participants

Youth are eligible to participate if all of the following criteria are satisfied at the time of randomisation:

- The youth is aged 14 to 20 years old.
- The youth has spent 5 years or less in Sweden.
- The youth arrived in Sweden unaccompanied.
- The youth screens positive on the Children’s Revised Impact of Event Scale (CRIES-8) PTSD screening tool (≥ 17 points).
- The youth is interested in participating in a group intervention.
- The youth consents to be randomised.
- The legal guardian consents to participation if the youth is < 15 years old.
- Caregiver contact details are provided if the youth is < 18 years old.
- There is no ongoing treatment where a therapist advises against participation in TRT.

### Recruitment

Youth (males and females) will be referred to the trial by community workers (e.g. nurses, social workers, NGO staff members) who have concerns about the youth’s mental health. Youth can also self-refer. The CRIES-8 [21] will be used to identify youth with symptoms of PTSD. Those with scores ≥17 will be offered participation in the study. The Child Health and Parenting (CHAP) research group at Uppsala University has established relationships with community sites with TRT-trained staff across Sweden (e.g. Huddinge, Linköping, Uppsala, Östersund, Stockholm, Västerås). New sites will be approached during the trial period. It is anticipated that around 10 sites will recruit to the trial. Information about the study will be distributed to community sites directly by CHAP and posted online on the CHAP website. In an exploratory study, 90% of those screened for participation met the cutoff on the CRIES-8 [28]. The treatment retention rate was 59%, with most dropouts occurring right before or just after the start of the group. To take these factors into account, an over-recruitment is planned for the present project.

Youth who meet the inclusion criteria will be invited to a group ‘information and assessment’ meeting. Written informed consent and pre-intervention measures will be collected by the research team at the meeting prior to randomisation on site. The informed consent relates only to the SUPpORT study; no ancillary studies are planned. No biological specimens will be collected as part of the SUPpORT study.

If a youth is randomised to the intervention group but does not attend the TRT sessions, he/she will remain in the research study and will be contacted at the data collection points. The youth (and legal guardian if the youth is < 15 years) will be informed of his/her right to withdraw from the research study. Youth will be informed that they can withdraw at any time and do not need to give a reason, and there will be no negative outcomes from withdrawing. No further data will be collected for withdrawn cases. All existing data will be retained unless a youth/legal guardian also asks for it to be removed (youth/legal guardians will be informed that this is possible up to the point that the data is analysed). Youth will be able to receive the TRT programme regardless of whether they withdraw their involvement in the research study.

In order to minimise attrition, the trial has been branded as the SUPpORT project (Swedish UnaccomPanied yOuth Refugee Trial); participants will be made aware of what involvement in the project entails from the outset; youth will be offered an incentive (shopping vouchers valued at 100SEK) at each data collection session to compensate for their time; and the research team will work to keep community contacts involved in the trial and help them to make referrals.

### Sample size

Recruitment of 218 eligible youth to the project (109 per trial arm) will allow detection of an effect size of 0.5 at *p* < 0.05 with 80% power and allows for a study dropout rate of up to 41%, as informed by an exploratory study (an effect size of 0.5 requires a minimum sample size of 64 participants per trial arm).

### Randomisation

A computer-generated randomisation sequence will be used to assign the participants to the intervention and waitlist-control arms in a 1:1 ratio. Block randomisation of block sizes 4 or 6 will be generated in a computerised randomisation schedule. Randomisation will take place after pre-intervention data collection. The allocation sequence will be concealed using an online central randomisation service set up and maintained by a professional third party (www.sealedenvelope.com) that will conceal the sequence until group assignment. The randomisation process will require the research team to either (1) log into a password-protected website or (2) send a Short Message Service (SMS) message and enter the relevant data of each newly recruited participant in order to receive the allocation.

### Blinding

Randomisation will take place in the community, at scheduled group ‘information and assessment’ meetings, directly after pre-intervention data collection. The research team will oversee the randomisation process. Participants will not be blinded to group allocation; they will be informed of group allocations immediately at the group meeting, along with local TRT group leaders. Allocation status will be recorded on a secure online platform (www.sealedenvelope.com). Data collection will not be blinded; however, as the outcome data is collected using self-completion questionnaires rather than through observation or interview, outcome data is less susceptible to information bias and interviewer effects [9]. Given that neither the participants nor group leaders are blinded, there is no requirement for an unblinding procedure. Outcome data spreadsheets will use anonymous participant identity numbers; however, group status will be apparent due to the inclusion of attendance data for the intervention group.

### Control arm

Youth assigned to the waitlist-control arm will receive services as usual because the aim of the trial is to determine whether the TRT programme provides added value. The offer is likely to include school health services and contact with their general practioner (GP). Other services are unlikely to be highly similar to the TRT programme, as reconnaissance suggests that typically few group therapy programmes are available for unaccompanied refugee youth. Any services that youth do receive, including other therapy programmes, will be captured in the Trimbos/institute for Medical Technology Assessment (iMTA) Questionnaire for Costs associated with Psychiatric Illness (TiC-P) (see Other measures section).

### Intervention arm

The intervention arm will receive the Swedish translation of the TRT programme and may access services as usual. The intervention uses TF-CBT as its foundation, agreed to be the method of choice for treating PTSD in children and adolescents [15]. The group-based cognitive behavioural programme includes five youth sessions and two caregiver sessions. A ‘getting to know each other session’ will be offered prior to the core TRT sessions and a ‘follow-up session’, which consolidates learning and enables participants to talk about their experience of taking part in the programme, will be offered afterwards. Sessions will be delivered over 7 consecutive weeks. Each session will last 2 h (including a break). For unaccompanied refugee youth, the ‘caregiver’ is a nominated adult (e.g. their legal guardian, an adult from their family home or a school counsellor). The sessions for youth incorporate several components of TF-CBT, including psychoeducation, affective modulation skills, cognitive coping and processing, trauma narrative, overcoming trauma reminders and future development. The caregiver sessions include an introduction to the TRT method and an overview of the content in the youth sessions. The caregivers are instructed in how to support the youth through listening and comforting, when needed, as well as through maintaining routines and activities. Additionally, the caregivers receive information on how to seek care if the youth needs additional help after TRT. Caregiver sessions are delivered without the youth, in parallel with the first two youth sessions. TRT group leaders receive 3 days of training in programme delivery from BRIS (Children’s Rights in Society). Two group leaders deliver each group (with the assistance of an interpreter, if necessary).

### Participant timeline

**Fig. 2:**
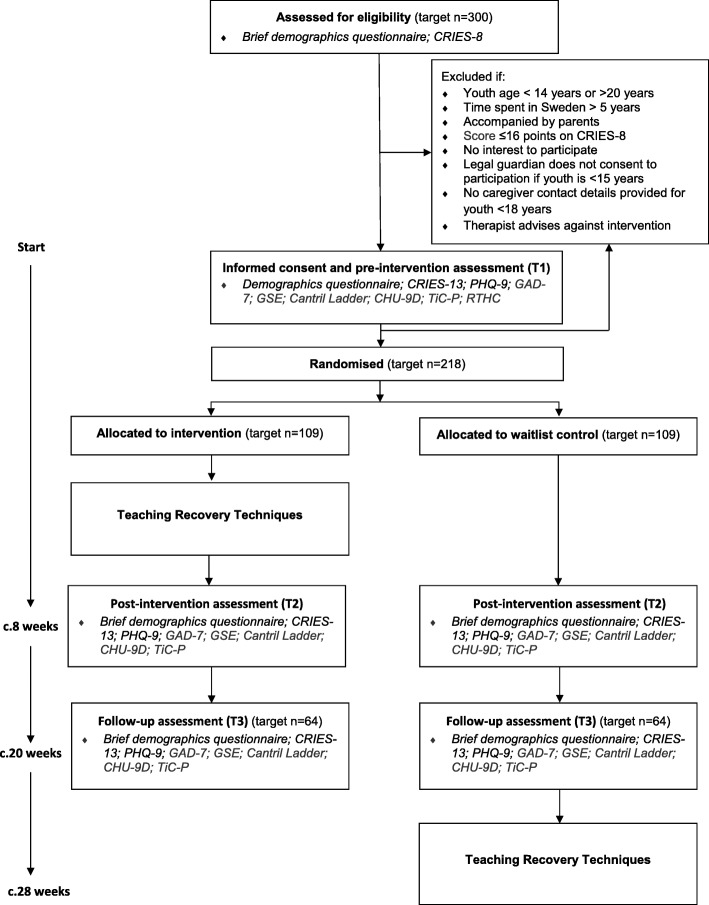
SUPpORT participant flow chart

A schedule of enrolment, interventions and assessments is shown in Fig. 1 and a flow diagram of the participant timeline is shown in Fig. 2. Youth are screened for eligibility, which could be done individually or at a group ‘information and screening’ meeting. For eligible youth, informed consent and pre-intervention assessments take place at a group ‘information and assessment’ meeting. A case will be randomised once the participant has completed all pre-intervention data collection. Follow-up data will be collected from all participants at scheduled group meetings at two points: first (T2), about 8 weeks (+/− 1 week) after randomisation (equivalent to end of TRT programme delivery), and second (T3), about 20 weeks (+/− 2 weeks) after randomisation (equivalent to about 3 months after TRT programme delivery).

### Outcome measures

The study will primarily measure changes in youth self-reported mental health, specifically symptoms of PTSD, depression and anxiety. A combination of primary mental health measures is being used due to the complex trauma that can be experienced by the youth. Apart from adverse events before and during migration, the asylum and resettlement process per se involves stressors. Complex trauma can lead to social difficulties, behavioural and emotional symptoms, psychosomatic problems and sleep problems as well as PTSD symptoms [11, 22, 33].

Secondary assessments will include measures of self-efficacy and well-being, both of which relate to the TRT programme theory of change. All outcome measures will initially be available in Swedish, English, Arabic, Dari, Farsi, Somali and Tigrinya, with other languages made available if necessary. The measures will be administered pre-intervention (T1), after intervention delivery (T2) and a few months later (T3). The specific metrics, methods of aggregation and time points for the outcomes are described in the Statistical methods section.

#### Children’s Revised Impact of Event Scale (CRIES-13)

The CRIES-13 [21] is a 13-item self-report measure of PTSD symptoms. Individual items are rated according to the frequency of their occurrence during the past week (None = 0, Rarely = 1, Sometimes = 3, and A lot = 5) and in relation to a specific traumatic event. Scores are obtained for 4 intrusion items (e.g. *Do you think about it even when you don’t mean to?*), 4 avoidance items (e.g. *Do you try not to talk about it?*) and 5 arousal items (e.g. *Do you get easily irritable?*). Total scores on the scale range from 0 to 65 with a cutoff score of 30 or above. The total score has been shown to have good internal consistency, and to successfully categorise more than 75% of children with and without a PTSD diagnosis [21, 42]. In a study of asylum-seekers in Sweden, the CRIES-8, a shorter version of CRIES which only includes the intrusion and avoidance items, was shown to have good internal consistency (Cronbach’s α = 0.75), and its factor structure was confirmed [27].

#### Patient Health Questionnaire-9 (PHQ-9)

The PHQ-9 [16] is a 9-item self-report instrument for screening, diagnosing, monitoring and measuring severity of depression. Individual items (e.g. *Little interest or pleasure in doing things*) are rated according to the frequency of their occurrence during the past 2 weeks (Not at all = 0, Several days = 1, More than half the days = 2, Nearly every day = 3). Total scores on the scale range from 0 to 27 with cutoff scores of 5, 10, 15 and 20 for mild, moderate, moderately severe and severe symptoms respectively. The instrument has shown high internal consistency, with Cronbach’s α = 0.86 and 0.89 in two primary care samples respectively, and test-retest reliability *r* = 0.84 [16]. Both construct validity and diagnostic validity for major depression have been established in several studies, and sufficient sensitivity (0.71–0.87) and high specificity (0.88–0.95) have been found for PHQ-9 ≥ 10 [12]. PHQ-9 has also been shown to be responsive in measuring treatment outcomes, and a change in scores of 5 has been suggested to reflect a clinically relevant change [18].

#### Generalised Anxiety Disorder-7 (GAD-7)

The GAD-7 [35] is a 7-item self-report measure originally developed to screen for generalised anxiety disorder. It has, however, also frequently been used to assess severity of more general anxiety symptoms [17]. Individual items (e.g. *Feeling nervous, anxious, or on edge*) are rated according to the frequency of their occurrence during the past 2 weeks (Not at all = 0, Several days = 1, More than half the days = 2, Nearly every day = 3). Total scores on the scale range from 0 to 21 with cutoff scores of 5, 10 and 15 for mild, moderate and severe symptoms respectively. It has shown high internal consistency (Cronbach’s α = 0.92) and seems to function well as an indicator of symptom severity [35].

#### General Self-Efficacy Scale (GSE)

The GSE [30] is a 10-item self-report measure that assesses the strength of individuals’ beliefs in their own ability to respond to difficult situations and to deal with obstacles or setbacks. Individual items (e.g. *I can always manage to solve difficult problems if I try hard enough*) are rated according to how true the statement is for that individual (1 = Not at all true, 2 = Hardly true, 3 = Moderately true, 4 = Exactly true). Total scores range from 10 to 40 with a higher score indicating more self-efficacy. In samples from 25 nations, Cronbach’s α ranged from 0.75 to 0.91 [29].

#### The Cantril Ladder

The Cantril Ladder [6] measures well-being and life satisfaction. The respondents are presented with a picture of a ladder numbered from 0 to 10, where the bottom of the ladder (0) represents their worst possible life and the top (10) represents the best. Respondents are asked to think about their life right now and place themselves on the ladder. Total scores range from 0 to 10 with a higher score indicating greater well-being and life satisfaction. A score of 4 or below is indicative of ‘suffering’ and 7 or above ‘thriving’. The scale has proven a valid measure of general psychosocial health among children/youth of ages 10–17 years [19]. The Cantril Ladder will be administered at each TRT session to inform a safety protocol. The total score will be used as a secondary outcome, assessed at T1, T2 and T3.

### Other measures

Basic demographic information and trauma history will be collected for all participants. Health-related quality of life and service consumption will be measured to inform the economic evaluation. A suicidality screening tool will be utilised as part of a safety protocol for participants who indicate they have had thoughts they would be better off dead (ninth item on PHQ-9) or ‘suffering’ on the Cantril Ladder (i.e. a score of 4 or below).

#### Demographics questionnaire

The study will use a short questionnaire to gather demographic information about the youth and his/her family. It includes variables such as youth age, gender, ethnicity, time spent in Sweden and asylum status. This data will be used to describe the sample, examine the extent to which demographic characteristics are balanced between trial arms, carry out attrition analyses (i.e. the extent to which participants who drop out from the intervention and waitlist-control arms are different on variables such as gender and ethnicity) and identify subgroups. The demographics questionnaire will be administered pre-intervention (T1). A brief version of the questionnaire that includes items for which the response may change (e.g. asylum status) will be administered after intervention delivery (T2) and a few months later (T3).

#### Refugee Trauma History Checklist (RTHC)

The RTHC [31] is a self-report measure of the occurrence of potentially traumatic experiences. It consists of 2 × 8 items, concerning potentially traumatic experiences that occurred before and during the respondent’s flight respectively. Results show low item non-response and adequate psychometric properties [31]. These data will be used to describe the sample, examine the extent to which potentially traumatic experiences are balanced between trial arms, carry out attrition analyses (i.e. the extent to which participants who drop out from the intervention and waitlist-control arms report different experiences) and identify subgroups. The RTHC will be administered pre-intervention (T1).

#### Child Health Utility 9D (CHU-9D)

The CHU-9D [36] is a self-report measure of health-related quality of life. It consists of 9 dimensions (worry, sadness, pain, tiredness, annoyance, school, sleep, daily routine and activities). Individual items are scored according to severity on the day from 1 (no problems) to 5 (severe problems). Originally developed for application with children aged 7–11 years [37–39], its practicality and validity in adolescents aged 11–17 years has also been demonstrated [7, 26]. In this study, responses to the CHU-9D will be scored using the UK scoring algorithm (the only available European algorithm). The scoring algorithm was generated on a utility scale and ranges from 0 for the worst health state to 1.0 for the best health state. These scores will be used to generate quality-adjusted life years (QALYs) over the trial period, and will inform the economic evaluation. The CHU-9D will be administered pre-intervention (T1), after intervention delivery (T2) and a few months later (T3).

#### Trimbos/institute for Medical Technology Assessment (iMTA) Questionnaire for Costs associated with Psychiatric Illness (TiC-P) Child and adolescent version

The TiC-P is a self-report measure of resource use for people with a psychiatric disorder. It is a generic questionnaire, meaning that the items are not related to a target disorder. Distinguishing between health care consumption and productivity losses as a consequence of the target disorder and comorbidity is difficult, especially in psychiatric disorders, as patients also may have physical symptoms that are connected to the psychiatric illness. Moreover, psychiatric comorbidity is a common occurrence in psychiatric illness. The TiC-P will ask about service contacts and absence from school/work over a time period preceding the date of the data collection. It will be administered pre-intervention (T1), after intervention delivery (T2) and a few months later (T3) and will inform the economic evaluation.

#### Columbia-Suicide Severity Rating Scale (C-SSRS) Screen Version

The C-SSRS Screen Version [24] is a 6-item structured interview or self-report measure that assesses the presence and severity of suicidal ideation and behaviour. Individual items (e.g. *Have you wished you were dead or wished you could go to sleep and not wake up?*) are rated according to presence over the past month (Yes or No). A positive response to item 3 (*Have you been thinking about how you might do this?*) indicates a moderate risk. A positive response to item 4 (*Have you had these thoughts and had some intention of acting on them?*), 5 (*Have you started to work out or worked out the details of how to kill yourself? Do you intend to carry out this plan?*) or 6 (*Have you ever done anything, started to do anything, or prepared to do anything to end your life?*) indicates a high risk. A three-site study including both adults and adolescents showed strong convergent validity with other established scales measuring suicidal ideation and attempts [24]. Cronbach’s α varied between 0.95 for intensity of suicidal ideation during the past week and 0.73 across all visits. The C-SSRS will be utilised as part of a safety protocol for participants who indicate they have had thoughts they would be better off dead (ninth item on PHQ-9) or ‘suffering’ on the Cantril Ladder (i.e. a score of 4 or below). The safety protocol details when and where to signpost or refer to another service and has been adapted to align with service availability in local areas. Safety protocol utilisation will be captured on the fidelity checklist (see the following discussion). Frequency of safety protocol use will be reported. Any spontaneously reported adverse events will be recorded and managed accordingly by a trained member of the trial team (medical, clinical psychology and psychiatry expertise on the team).

### Intervention fidelity

A fidelity-monitoring tool has been developed by the CHAP research team in association with TRT group leaders in order to promote and monitor adherence to the core design of the programme. The fidelity-monitoring process will be implemented by TRT group leaders, who will share the data with CHAP for research purposes. After each TRT session, the group leaders complete a self-report adherence checklist, which captures group leader details; number of participants; use of interpreter(s); number of people who required the safety protocol; and the range of core components delivered. Session attendance lists will also be shared with CHAP to inform individual participant dose.

### Data collection

Pre-intervention data collection and randomisation is planned to take place between January 2019 and February 2021. End of TRT programme delivery data collection occurs about 8 weeks post pre-intervention data collection, and is therefore projected to take place between April 2019 and May 2021. Endpoint data collection occurs about 20 weeks after pre-intervention data collection and is projected to take place between July 2019 and September 2021.

Outcome data will be collected using a secure online platform (Qualtrics). TRT facilitators will be given the option of submitting fidelity data online or on paper forms. Data will be exported/inputted into an SPSS file for analysis. Anonymous participant identity numbers will be used. The file will be saved on the university server, which is automatically backed up. All procedures comply with current regulations on personal data management.

The youth (and legal guardian if the youth is < 15 years) will be informed that the data provided will be treated confidentially. He/she will be made aware that in published reports the results will be reported anonymously and at a group level, meaning that it will not be possible to identify any individual or attribute any information to them. Participants will be informed that if they disclose anything concerning their personal safety, then a safety protocol will be implemented.

### Statistical methods

Baseline and demographic characteristics will be summarised using means and standard deviations (or medians and interquartile ranges) for continuous variables and percentages for categorical variables. A set of strategies will be employed to minimise the amount of missing data (e.g. offering incentives for completing follow-ups). Reasons for dropouts for each condition will be reported. The possible impact of missing data will be examined via sensitivity analyses of augmented data sets. Including the dropouts and participants with missing data will be made possible by using modern analytical methods.

The comparison of the trial arms will use an intention-to-treat framework with participants analysed according to the trial arm to which they were randomised, regardless of whether or not they received the intervention. Linear mixed models will be used to compare outcomes for the trial arms. The primary outcome is total score group differences on the mental health outcome measures (i.e. CRIES-13, PHQ-9, GAD-7) after programme delivery (T2). The secondary outcomes are mental health outcome measures (i.e. CRIES-13, PHQ-9, GAD-7) total scores at endpoint (T3) and GSE and Cantril Ladder total scores after programme delivery and at endpoint (T2 and T3).

For the mental health outcome measures (CRIES-13, PHQ-9,; GAD-7), participants will also be classified as ‘recovered’, ‘improved’, ‘unchanged’ or ‘deteriorated’ based on the Reliable Change Index (RCI) and Clinically Significant Change (CSC) approach [10, 13]. This approach incorporates both a measure of whether the change in scores is larger than what is expected due to outcome measure reliability as well as the participant’s shift from a clinical state to a non-clinical state. The proportions of classifications will be compared across the trial arms.

Fidelity to the design of the intervention will be summarised using descriptive statistics. It will be assessed in terms of the different dimensions measured (adherence and dose). A secondary analysis will be undertaken to quantify the extent to which the intervention effect on the primary outcomes is determined by participation in the intervention (number of sessions received). Further moderation analyses will examine the associations between improvement status and participants’ characteristics (e.g. age, gender, suicidal ideation).

For the economic evaluation, the outcomes and costs between the intervention and control groups will be compared using generalised linear models (GLMs), which allows the consideration of other distributions and functional forms to fit the data [2]. Two types of evaluation will be conducted: (1) a cost-utility analysis with outcomes measured in QALYs and (2) a cost-effectiveness analysis with proportion of participants classed as treatment success expressed as incremental cost-effectiveness ratios [8]. The cost-effectiveness ratios describe (1) the price for one additional QALY, i.e. one life year with full health, and (2) the price to get an additional successfully treated participant.

## Discussion

Challenges are anticipated, including a high level of attrition and poor literacy among participants combined with a reliance on self-report measures. However, the trial has been designed to mitigate these challenges where possible (e.g. with over-recruitment and retention strategies) and will be instrumental in building the Swedish evidence base for refugee youth mental health interventions. In particular, it will examine the impact of a brief therapy programme (weekly sessions over 7 weeks) with unaccompanied refugee youth who are reporting symptoms of PTSD. The project also provides an opportunity to demonstrate that randomised controlled designs can be used to evaluate social interventions in real-world, community settings.

### Trial status

The protocol is version 2 (11th October 2019). Recruitment efforts began in January 2019 and randomisation began on 8th April 2019. Recruitment will continue until February 2020.

## Supplementary information

**Additional file 1.** SUPpORT SPIRIT checklist.

## Data Availability

The results from the SUPpORT trial are due to be submitted for publication in September 2021. Authorship will be granted for substantive contributions to the design, conduct, interpretation and reporting of the SUPpORT trial; the ultimate decision on authorship will be made by the Principal Investigator (AS). Publications will be open access. The data sets generated during the current study will be available from the corresponding author on reasonable request.

## Abbreviations

CHAP: Child Health and Parenting (research group)
CHU-9D: Child Health Utility 9D
CRIES: Children’s Revised Impact of Event Scale
CSC: Clinically Significant Change
C-SSRS: Columbia-Suicide Severity Rating Scale
GAD-7: Generalised Anxiety Disorder 7 item (scale)
GSE: General Self-Efficacy Scale
NGO: Non-governmental organisation
PHQ-9: Patient Health Questionnaire-9
PTSD: Post-traumatic stress disorder
QALY: Quality-adjusted life year
RCI: Reliable Change Index
RCT: Randomised controlled trial
SMS: Short Message Service (text message)
TiC-P: Trimbos/institute for Medical Technology Assessment Questionnaire for Costs associated with Psychiatric Illness
TRT: Teaching Recovery Techniques
URM: Unaccompanied refugee minor
